# A CMOS Humidity Sensor for Passive RFID Sensing Applications

**DOI:** 10.3390/s140508728

**Published:** 2014-05-16

**Authors:** Fangming Deng, Yigang He, Chaolong Zhang, Wei Feng

**Affiliations:** 1 School of Electrical Engineering and Automation, Hefei University of Technology, Hefei 230009, China; E-Mails: 18655136887@163.com (Y.H.); zhangcl@aqtc.edu.cn (C.Z.); Fengw1981@126.com (W.F.); 2 School of Electrical and Electronic Engineering, East China JiaoTong University, Nanchang 330013, China

**Keywords:** RFID technology, passive RFID tag, humidity sensor, interface circuit, phase-locked loop, CMOS technology

## Abstract

This paper presents a low-cost low-power CMOS humidity sensor for passive RFID sensing applications. The humidity sensing element is implemented in standard CMOS technology without any further post-processing, which results in low fabrication costs. The interface of this humidity sensor employs a PLL-based architecture transferring sensor signal processing from the voltage domain to the frequency domain. Therefore this architecture allows the use of a fully digital circuit, which can operate on ultra-low supply voltage and thus achieves low-power consumption. The proposed humidity sensor has been fabricated in the TSMC 0.18 μm CMOS process. The measurements show this humidity sensor exhibits excellent linearity and stability within the relative humidity range. The sensor interface circuit consumes only 1.05 μW at 0.5 V supply voltage and reduces it at least by an order of magnitude compared to previous designs.

## 1. Introduction

Radio Frequency IDentification (RFID), as a wireless automatic identification technology, is widely applied in traffic management, logistics transportation, medicine management, food production, *etc*. [1]. Passive RFID tags offer several advantages such as battery-less operation, wireless communication, high flexibility, low cost and fast deployment, which all result in their extensive applications in commercial use [2]. The passive RFID tag uses the wireless energy from the RFID reader as its power supply. Hence, the power dissipation of the passive RFID tag, which determines the maximum reading distance of the RFID reader, is crucial for the design of passive RFID tags. Furthermore, limited by the rectifier efficiency, the internal circuits of passive RFID tags have to operate on a supply voltage which is always lower than 1 V. Recently, with the rapid developments of the Internet of Things and sensor technology, research on adding sensing functionality to RFID tags has become a hot topic [3–6]. These smart RFID sensing tags not only extend the fields of application of RFID, but also contribute to reduce the cost of RFID system fabrication. Therefore, the design of these sensors for passive RFID applications has to meet the requirements of low-voltage and low-power.

Humidity measurements are essential for a wide range of applications, including environment monitoring systems, process control systems, pharmaceutical and biomedical applications, food preservation, *etc*. [7]. Humidity sensors usually measure Relative Humidity (RH) rather than absolute humidity. Relative humidity is the ratio of the moisture level to the saturated moisture level at the same temperature and pressure and expressed as a percentage. Capacitance [8] and resistance [9] are the two main measured parameters and the majority of commercial humidity sensors are of the capacitive type, which can offer low power consumption and wide operating range. Furthermore, they requires less complex interface circuits compared with the resistive type.

Various materials, such as porous ceramics and hygroscopic polymers, are used as humidity-sensitive materials [10]. Because of its good moisture absorption and compatibility with integrated circuit (IC) fabrication technologies, polyimide is a good candidate for moisture sensing films in capacitive humidity sensors for both Micro-Electro-Mechanical System (MEMS) and Complementary-Metal-Oxide Semiconductor (CMOS) technologies [11,12]. The humidity sensor fabricated in CMOS technology can easily be integrated with the readout circuits on a single chip [13,14], which has several advantages including improved accuracy, reduced size and lower fabrication cost. Some capacitive humidity sensors based on CMOS technology have been reported [15–19]. However, the designs [15–18] all require some post-processing steps and the design in [19] uses materials and steps not commonly found in standard fabrication processes. All of these factors will undoubtedly increase the fabrication costs.

CMOS technology has been mainly developed for digital IC. Benefitting from technology scaling, digital IC achieves great improvements on speed, power dissipation, chip area, *etc*. However, analog IC is less scalable and suffers more when going to nanometer CMOS technology. Apart from the issues of matching and noise, the threshold voltage is reduced less remarkably compared to the power supply, resulting in a reduced voltage headroom for traditional analog amplitude-based sensor interfaces [20]. To cope with this challenge, a novel method transferring sensor signal processing from the traditional voltage domain to the frequency domain has been put forward in recent years [21,22]. This method allows the use of fully digital circuits rather than analog circuits, overcoming the limitation of the decreased voltage headroom. Hence the interface circuit with this approach can work with ultra-low supply voltages and is especially suitable for low power interface design.

The paper aims to present a low-cost low-power humidity sensor for RFID sensing applications. The rest of the paper is organized as follows: Section 2 presents a low-cost humidity sensor which is fabricated in standard CMOS technology without any further post-processing. Section 3 introduces the Phased-Locked Loop (PLL)-based sensor interface theory first and then illustrates the implementation of the fully-digital sensor interface in detail. The measurement results are presented and compared with previous designs in Section 4. Finally, some conclusions are drawn in Section 5.

## 2. Capacitive Humidity Sensing Element

**Figure 1.**
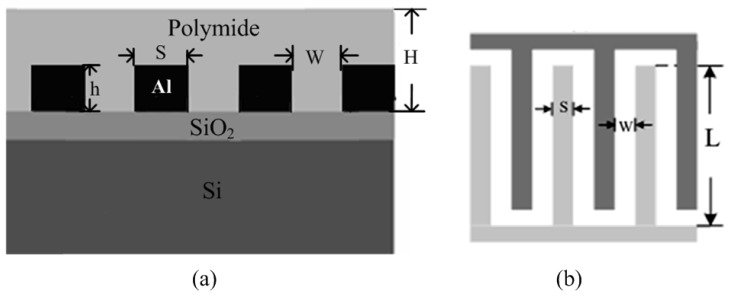
Proposed humidity sensor: (**a**) humidity sensor structure; (**b**) top view of the humidity sensor.

For a CMOS process, interdigitated top metal fingers, with polyimide filled into the finger gaps, can be utilized for capacitive humidity sensing. The line-to-line coupling capacitance of the top metal is sensitive to the dielectric constant of the filling material. Furthermore, due to the high precision of photolithography, such kinds of sensing structures are highly reproducible with less inter-die variations [23]. Figure 1a illustrates the structure of the proposed capacitive humidity sensor. It begins with the thermal growth of a thick SiO_2_ layer on the Si wafer. A thick aluminum layer is then deposited and patterned with standard optical lithography and wet etching over the SiO_2_ in order to form the interdigitated structure. The sensing film is polyimide that is coated on the interdigitated electrodes. In order for moist air to access the sensing film, there must be a large area of the polyimide exposed to the air. As is seen from Figure 1b, *L* is the length of aluminum electrodes, *S* is the width of each electrode, and *W* is the distance between adjacent electrodes. The thickness of the polyimide layer is *H*, which is generally larger than the metal thickness *h*.

The dielectric constant of the sensing film can be expressed by the Looyenga empirical equation [24] as follows:

(1) $${ɛ}_{\text{wet}}={\left[\left(\gamma \left({ɛ}_{\text{water}}^{⅓}-{ɛ}_{\text{dry}}^{⅓}\right)+{ɛ}_{\text{dry}}^{⅓}\right)\right]}^{3}$$

Where *ε_wet_* represents the dielectric constant of the polyimide film with absorbed water, *γ* is the fractional volume of water absorbed in polyimide, *ε_dry_* is the dry polyimide film dielectric constant, and *ε_water_* is the water dielectric constant. The relationship between *γ* and relative humidity can be modeled by Daubinin equation [25] as follows:

(2) $$\gamma =\gamma_{m}\phi \left(T\right)RH^{\beta \left(T\right)}$$

where *γ_m_* is maximum factional volume at T_0_=298K, *ф*(T) is the temperature dependence on the adsorption coefficient and *β*(T) is the temperature dependence of the relative dielectric constant of water and the catalytic effect. The dielectric constant of the polyimide film *ε_wet_* (and in turn the capacitance of the sensor) depends on the relative humidity, which increases or decreases when moisture penetrates or leaves the film. The mechanism for moisture absorption (or desorption) depends on the film properties (not sensor geometries) and determines the sensitivity of the sensor [26].

For a *N* finger array sensor, the total sensor capacitance can be expressed as [27]:

(3) $$C_{\text{sensor}}=N{ɛ}_{\text{wet}}\frac{Lh}{W}$$

The selection of the thickness of polyimide layer *H* has a great influence on the sensitivity and response time of the sensor. Generally, a humidity sensor with a thicker polyimide layer has better sensitivity and longer response time. Trading off the various factors, including sensitivity, response time, hysteresis performance and chip area, this work chooses *N* = 40, *L* = 200 μm, *W* = 2.5 μm, *S* = 2.5 μm, *h* = 1 μm and *H* ≈ 2 μm. The sensor capacitance can be evaluated in accordance with Equation (3) if the dielectric constant of the polyimide film *ε_wet_* is given.

## 3. Humidity Sensor Interface

### 3.1. PLL-Based Sensor Interface Theory

**Figure 2.**
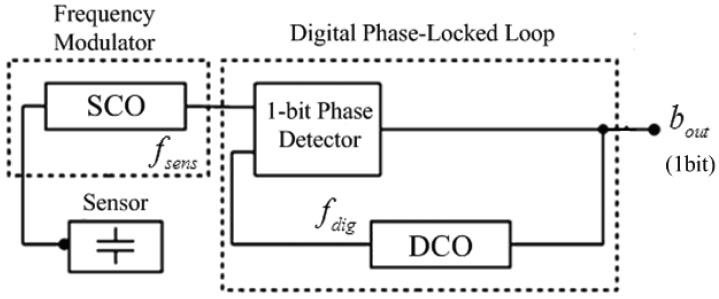
Architecture of PLL-based sensor interface.

The architecture of PLL-based sensor interface, developed from Danneels [21], is shown in Figure 2. It consists of two main blocks: a frequency-modulating block, which converts the sensor information to the frequency domain, and a frequency-demodulating block, which converts the frequency to the digital domain, resulting in a complete sensor-to-digital flow. The frequency-modulating block consists of a Sensor-Controlled Oscillator (SCO) and directly converts the capacitive value of the sensor to a corresponding frequency *f_sens_*. The frequency-demodulating block is a digital first-order Bang-Bang-Phased-Locked-Loop (BBPLL), consisting of a single-bit phase detector and a Digitally-Controlled Oscillator (DCO). This BBPLL measures whether *f_sens_* leads or lags *f_dig_* (the frequency of the DCO) and hence the DCO is only steered by a single-bit signal *b_out_*. When the entire feedback loop is locked, *f_sens_* shifts between a maximum and minimum value which corresponds to the maximal and minimal value of the sensor. The average digital frequency *f_dig_* will correspond to the sensor frequency *f_sens_*. Therefore, the over-sampled output *b_out_* represents the digital value of the sensor value.

This architecture employs the BBPLL block, which has advantages ranging from low voltage capabilities, low power consumption, small chip area, and scalability to smaller technologies and robustness to process variation [28,29]. Another significant improvement of this architecture is the direct conversion from capacitive sensor signal to the frequency domain, avoiding any intermediate transformation of the capacitive information to the voltage domain.

### 3.2. Implementation of Fully-Digital Capacitive Sensor Interface

**Figure 3.**
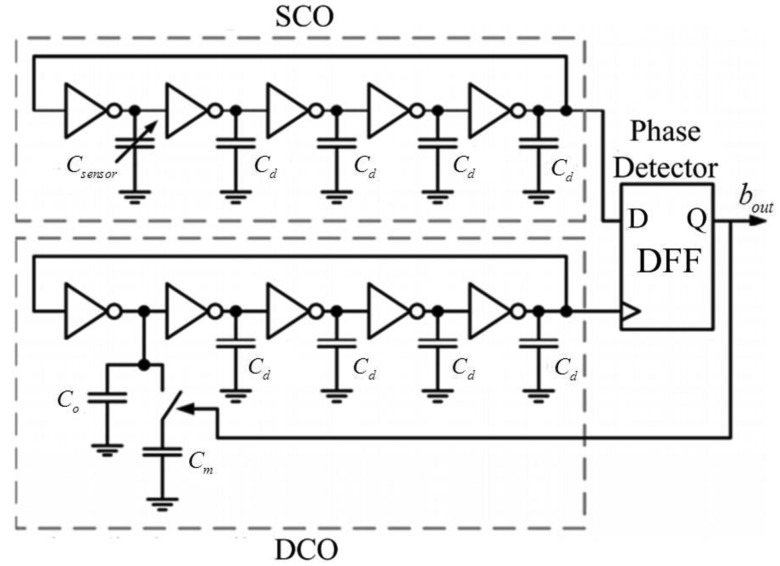
Proposed fully-digital capacitive sensor interface.

The implementation of the proposed fully-digital capacitive sensor interface is shown in Figure 3. Both the SCO and the DCO are implemented as five-stage inverter-based ring oscillators. The sensor capacitor *C_sensor_* acts as the variable load on a single stage of the SCO, thereby generating a sensor-controlled frequency *f_sens_*. The variable capacitive load on a single stage of the DCO consists of two capacitors, *C_o_* and *C_m_*. The capacitor *C_o_*, designed equal to the quiescent value of *C_sensor_*, is always connected to the DCO. But the capacitor *C_m_*, designed slightly larger than the maximum variation of *C_sensor_*, is swapped in or out of the DCO depending on the feedback from the single-bit phase-detector. Considering the issues of system linearity and process variation, *C_m_* is normally designed as a programmable capacitor. The dummy load capacitor *C_d_* at each stage should be carefully designed to be small enough for high resolution performance.

**Figure 4.**
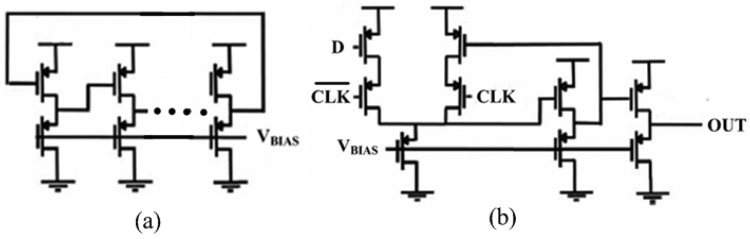
Detailed circuit of sensor interface: (**a**) ring oscillator circuit; (**b**) phase detector circuit.

As shown in Figure 4, the ring oscillator employs simple inverters and the phase detector is only a D-Flip-Flop (DFF), which both are implemented in PMOS-only logic for increased gain and swing. As we discussed above, when the entire feedback loop is stable, the average values over time of *f_sens_* and *f_dig_* are equal, thus the two frequency controls of the SCO and DCO are correlated. Since both oscillators are implemented identically, the control of the SCO, the sensor value, is correlated to the control of the DCO, the single-bit output of the phase detector. Hence the value of the sensor is digitized in this single-bit DCO control signal (*b_out_*). In order to increase resolution, *b_out_* is over-sampled by taking its average duty cycle overtime [29].

For this work, the humidity sensor's capacitance varies from 5–6.5 pF within the relative humidity range. Hence the capacitors *C_o_* and *C_m_* are selected as 5 pF and 2 pF, respectively. The dummy load capacitors (*C_o_*) at each stage are chosen to be 1 pF. Due to the fully digital architecture, the power supply of the interface is selected as 0.5 V, which is close to the process threshold voltage.

## 4. Measurement and Discussion

**Figure 5.**
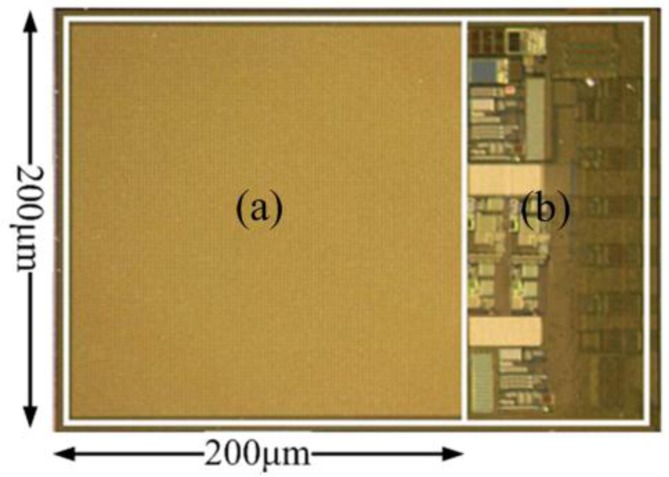
Microphotograph of the fabricated chip: (**a**) capacitive humidity sensor element; (**b**) interface circuit.

The proposed humidity sensor, fabricated in the Taiwan Semiconductor Manufacturing Company (TSMC) 0.18 μm 1P6M mixed-signal CMOS process, is shown in Figure 5. It includes two parts: the capacitive humidity sensor element (a) and the interface circuit (b), which cover 0.04 mm^2^ and 0.008 mm^2^, respectively. Two test humidity sensors, with 1 μm-thick and 2 μm-thick polyimide layers respectively, were measured in a Votsch VCL4003 (Votsch China, Taichang, China) temperature and climate test chamber.

**Figure 6.**
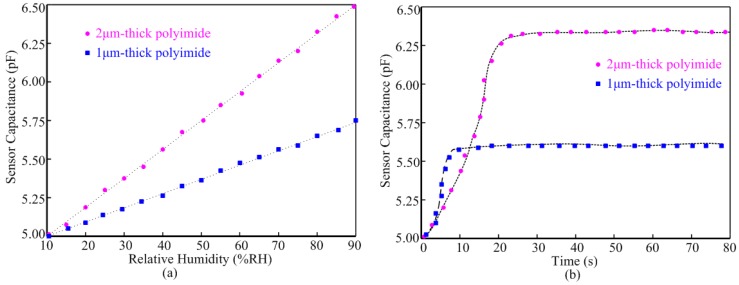
Performance comparison of the sensors with different polyimide layers at 25 °C; (**a**) sensitivity comparison; (**b**) response time comparison.

Figure 6 illustrates the polyimide thickness impacts on the performance of the humidity sensor at 25 °C. Figure 6a shows the sensor value with respect to the Relative Humidity (RH). Both sensors exhibit good linearity from 10%RH to 90%RH. The sensor with 2 μm-thick polyimide layer achieves 18.75 fF/%RH sensitivity, which is almost twice the sensitivity of the sensor with 1 μm-thick polyimide. The response time comparison of the two sensors is shown in Figure 6b. It was measured to 90% point of the final steady state capacitance after an abrupt relative humidity change from 10%RH to 80%RH at 25 °C. The response time of the sensor with 2 μm-thick polyimide layer is about 20 s, which is longer than the response time of the sensor with 1 μm-thick polyimide layer. For this work, the 20 s response time is acceptable and hence we coated the humidity sensor with 2 μm-thick polyimide for better sensitivity performance.

**Figure 7.**
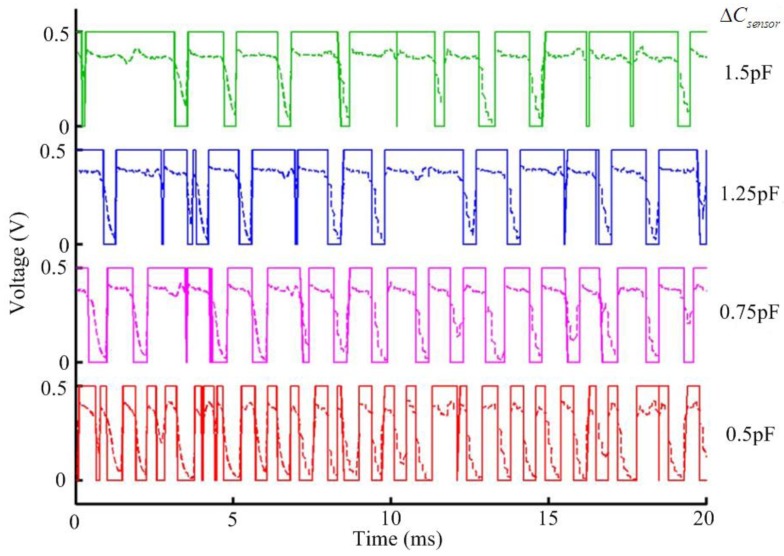
Measured outputs (dotted line) and digitized over-sampled outputs (solid line) for different Δ*C_sensor_* of the sensor with 2 μm-thick polyimide layer at 25 °C.

Figure 7 compares the different outputs corresponding to the different value change (Δ*C_sensor_*) of the humidity sensor with 2 μm-thick polyimide layer at 25 °C. The measured outputs (dotted line) and the digitized over-sampled outputs (solid line) are shown together. It is obvious that the average duty cycle of the output increases in proportion to the value of Δ*C_sensor_*.

**Figure 8.**
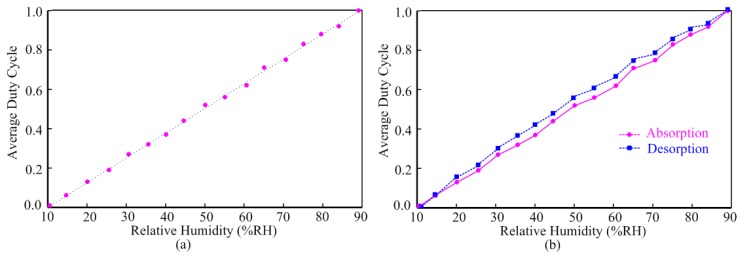
Sensitivity measurements of the humidity sensor: (**a**) linearity performance; (**b**) hysteresis performance.

The measured sensitivity of the humidity sensor is shown in Figure 8. Figure 8a illustrates the average duty cycle responses of the output with respect to the relative humidity at 25 °C. Within the relative humidity range, this humidity sensor achieves high linearity performance at 25 °C. As it is shown in Figure 8b, the humidity sensor exhibits a reduced hysteresis. The maximum difference between the moisture absorption and desorption at the point 55%RH is not exceeding 7%.

**Figure 9.**
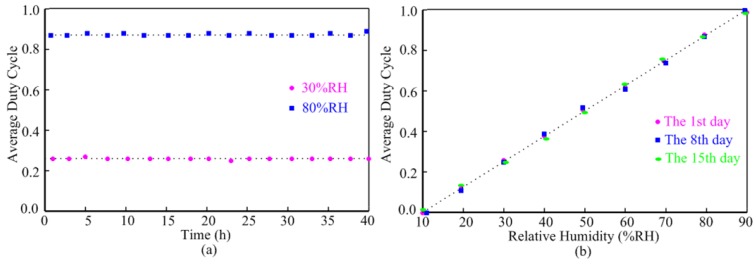
Stability measurement of the humidity sensor: (**a**) short-term stability; (**b**) long-term repeatability.

The stability measurement of the humidity sensor is given in Figure 9. The sensor, as shown in Figure 9a, was tested at the humidity of 30%RH and 80%RH for 40 h at 25 °C respectively and no obvious drift was observed. Figure 9b presents the test results of average duty cycle of interface outputs *versus* relative humidity on the 1st, 8th, 15th day so as to study the repeatability of the humidity sensor with time. It is obviously that the humidity sensor exhibits excellent repeatability.

Table 1 shows a comparison of the performance parameters of the proposed sensor interface with previous interface designs [30–33]. Owing to its simple architecture, the proposed sensor interface covers reduced chip area and can operate on 0.5 V ultra-low supply voltage. Despite the moderate Effective Number Of Bits (ENOB), this interface reduces power consumption at least one order of magnitude in respect with the previous designs.

## 5. Conclusions

The paper presents a integrated capacitive humidity sensor for RFID sensing applications. The humidity sensor is implemented in a standard CMOS process without any further post-processing, which results in low fabrication costs. The sensor interface, based on PLL architecture, transfers sensor signal processing from the traditional voltage domain to the frequency domain. It employs a fully-digital architecture and therefore can operate on an ultra-low supply voltage, which results in low power dissipation. The measurement results prove its excellent performance in the areas of linearity, stability, chip area and power dissipation. In particular the proposed sensor interface can work with 0.5 V supply voltage and consumes only 1.05 μW. Therefore this humidity sensor is particularly suitable for the mass-production of RFID sensing tags.

## Figures and Tables

**Table 1.** Performance Comparison of Integrated Sensor Interfaces.

**Interface**	**Technology (μm)**	**V-Supply (V)**	**ENOB (bits)**	**Area (mm^2^)**	**Power (μW)**
This work	0.18	0.5	6.8	0.008	1.05
[30]	0.5	3	8	9.28	6.9
[31]	0.35	3.3	12.7	0.048	1440
[32]	0.16	1.2	12.5	0.28	10.3
[33]	0.32	3	12	0.52	84
