# Correction: Deng, F., *et al.* A CMOS Humidity Sensor for Passive RFID Sensing Applications. *Sensors* 2014, *14*, 8728–8739

**DOI:** 10.3390/s140713171

**Published:** 2014-07-22

**Authors:** Fangming Deng, Yigang He, Chaolong Zhang, Wei Feng

**Affiliations:** 1 School of Electrical Engineering and Automation, Hefei University of Technology, Hefei 230009, China; E-Mails: 18655136887@163.com (Y.H.); zhangcl@aqtc.edu.cn (C.Z.); Fengw1981@126.com (W.F.); 2 School of Electrical and Electronic Engineering, East China JiaoTong University, Nanchang 330013, China

The authors wish to make the following corrections to this paper [1]. Figures 2, 3 and 4b were adapted from reference [2]. The corrected Figure 7 should be,

The authors would like to apologize for any inconvenience caused to the readers by these changes.

## Figures and Tables

**Figure 7.**
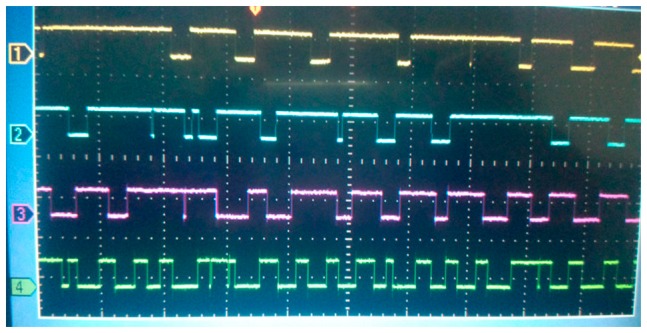
Measured outputs for different Δ*C_sensor_* of the sensor with 2 μm-thick polyimide layer at 25 °C.
